# Regional brain and cerebrovasculature morphology during normative aging in male and female C57BL/6N mice

**DOI:** 10.3389/fnagi.2026.1852741

**Published:** 2026-06-16

**Authors:** Amandine Jullienne, Tannoz Norouzi, Erik J Behringer, Andre Obenaus

**Affiliations:** 1 Department of Pediatrics, University of California Irvine, Irvine, CA, United States; 2 Division of Biomedical Sciences, School of Medicine, University of California Riverside, Riverside, CA, United States; 3 Department of Basic Sciences, Loma Linda University, Loma Linda, CA, United States

**Keywords:** brain morphology, cortex, lifespan, MRI, vessel painting, vessels, white matter

## Abstract

**Introduction:**

Normative aging is the process of gradual physical, cognitive and biological changes that occur with advancing age. This contrasts to pathological processes, such as neurodegeneration where changes are accelerated and more pronounced. Brain morphology and its cerebrovasculature are known to progressively alter during normative human and rodent lifespan.

**Methods:**

We assessed regional brain volumes (morphology) and cerebrovasculature characteristics across three lifespan epochs (6, 18, and 26 months of age) in male and female C57BL/6N mice, a widely used strain. The cerebrovasculature was assessed in mice using a vessel staining method we call vessel painting. High resolution magnetic resonance imaging (MRI) was acquired *ex vivo* to examine whole brain and regional volumes.

**Results:**

While no overt changes in cortical vasculature were detected across age or sex, we observed age-related changes in total and regional brain volumes. Selected brain regions exhibited sex-specific alterations.

**Conclusion:**

Our study reports regional brain changes across the lifespan in an age- and sex-dependent manner in C57BL/6N mice.

## 1 Introduction

With increasing life expectancy there comes increased risk of disease, dementia and disability (Brown, 2015). Advances in neuroimaging techniques such as magnetic resonance imaging (MRI) have provided us with an unprecedented ability to map and study healthy, aging, and diseased brain in a non-invasive manner, providing unique insights (Hagiwara et al., 2025). Human healthy/normative aging noted grey and white matter loss associated with increased ventricular volumes (Scahill et al., 2003; Salat et al., 2004; Abe et al., 2008; Hutton et al., 2009; Aljondi et al., 2019; Riddle and Taylor, 2020; Bethlehem et al., 2022). These progressive aging attributes in healthy individuals have also been reported in a variety of neurodegenerative disorders where they are often more pronounced compared to age-matched healthy subjects (Fjell et al., 2014). For example, brain atrophy and enlarged ventricles are also found in Alzheimer’s disease (Jack et al., 1997; Woodward et al., 2024), multiple sclerosis (Chen et al., 2025), and Huntington’s disease (Georgiou-Karistianis et al., 2013). Growing evidence suggests that brain alterations during aging may be linked to cerebrovascular deficits (Bullitt et al., 2010), which are known to be involved in Alzheimer’s disease (Uh et al., 2010; Zlokovic, 2011; Halvorsen et al., 2025). Normative aging is linked with decreased cerebral blood flow and vessel numbers, and with increased vessel tortuosity (Bullitt et al., 2010; Aanerud et al., 2017; Xu et al., 2017; Deshpande et al., 2022; Zhang et al., 2022).

Clinical evidence has identified major sex differences in the human brain. Brain volumes are larger in men than women, although males exhibit increased variance (Cosgrove et al., 2007; Guma et al., 2024). Men have a larger amygdala and hypothalamus but their hippocampi and caudate are smaller than females (Cosgrove et al., 2007; Guma et al., 2024). Human brain aging sex differences have been observed with significantly higher brain volume loss and ventricle enlargement in men compared to women (Armstrong et al., 2019). The cerebrovascular system in women has a higher global cerebral blood flow (Cosgrove et al., 2007) at least until 65 years of age (Aanerud et al., 2017). In a magnetic resonance angiography study examining healthy volunteers, intracerebral blood vessels radii were consistently found to be larger in men compared to women from 30 to 70 years of age (Bullitt et al., 2010).

Rodents, and especially mice, are extensively used in aging and neurodegenerative studies because of their relatively short lifespan and the ability to readily modify their genes. In clinical and preclinical studies, it is essential to understand how brain morphology and vasculature change with age and biological sex to allow for the distinction between pathology and healthy aging. Only a few studies have assessed brain region volumes across the healthy rodent lifespan, mostly focused on male rats (Mengler et al., 2014; Alexander et al., 2020) and male mice (Clifford et al., 2023). Few studies have analyzed male and female rats (Zhao and Zahr, 2025) or mice (Raikes et al., 2025). In contrast to human brains which exhibit decreased volumes after adolescence (Courchesne et al., 2000; Bethlehem et al., 2022), mouse brains continue to increase in size with normative aging (von Kienlin et al., 2005; Maheswaran et al., 2009; Jullienne et al., 2023), with no overt sex differences (Guma et al., 2024). The cerebral vasculature in the aging mouse exhibits decreased vessel density and increased tortuosity in alignment with similar alterations in humans (Lowerison et al., 2022; Bennett et al., 2024).

In the present study we examined global and regional brain volumes using high resolution MRI followed by quantification of cortical surface cerebrovascular morphology in a cohort of adult C57BL/6N mice. We examined sex differences at three distinct ages: mature (6 months), late middle-age (18 months), and old (26 months). These mouse ages have been approximated to human ages where a 6-month-old mouse is considered equivalent to 30 human years, an 18-month-old mouse is equivalent to 50 human years, and a 26-month-old mouse is equivalent to 70–80 human years (Flurkey et al., 2007). We acknowledge that mouse to human assessments depend on the features of interest (Semple et al., 2013; Agoston et al., 2019). Our goal was to provide a baseline of regional brain volumes, including those vulnerable to aging pathology, and cortical surface angioarchitecture that could provide a benchmark for future studies using mouse models of neurodegenerative disorders. We report age-related changes in total and regional brain volumes, with sex differences (total cortex, piriform area, amygdala, cingulum), and other regions only changed in males (motor and retrosplenial cortices) or in females (whole brain, cerebrum, CA1, total white matter, corpus callosum). We found no significant cross-sectional differences between males and females within each age group. Interestingly, we also observed no sex differences in cortical surface vasculature across the lifespan.

## 2 Materials and methods

### 2.1 Animals

All experiments were carried out in accordance with the Institutional Animal Care and Use Committee at the University of California, Irvine, consistent with Federal guidelines and following ARRIVE guidelines (Percie du Sert et al., 2020). Male and female C57BL/6N mice (RRID:MGI:2159965, Charles River Laboratories/National Institute on Aging) were divided into three different age groups: mature (6 months), late middle-age (18 months), and old (26 months), with N = 10 mice/age/sex. All animals were kept in a temperature-controlled facility with a 12-h light/dark cycle. Animals had unlimited access to food and water while being group-housed. All animals underwent vessel painting perfusion and *ex vivo* magnetic resonance imaging (MRI).

### 2.2 Vessel painting and vascular analysis

Vessel painting was performed as previously described (Salehi et al., 2018). This technique is based on the ability of the lipophilic fluorescent dye 1,1′-dioctadecyl-3,3,3′3’-tetramethylindocarbocyanine perchlorate (DiI, Life Technologies, Carlsbad, CA, USA) to bind to lipid membranes, allowing for the staining of the vasculature. Briefly, mice were anesthetized with Ketamine/Xylazine (90/10 mg/kg) and 500 μL of DiI (0.3 mg/mL in PBS containing 4% dextrose) was injected into the left cardiac ventricle prior to perfusing with 20 mL of phosphate-buffered saline (PBS) and 10 mL of 4% paraformaldehyde (PFA). Mouse heads were post-fixed in 4% PFA for 24 h then washed and stored in PBS until *ex vivo* MRI.

After MRI acquisition was completed, brains were carefully extracted from the cranial vault, and a wide-field fluorescence microscope (RRID:SCR_025160, Keyence Corp, Osaka, Japan) was used to image stained brains. Axial images were acquired with a 2X objective and processed as we have described (Salehi et al., 2018) using the Angiotool software (RRID:SCR_016393; Zudaire et al., 2011) which extracts vessel morphometric and spatial features including vessel density, number of junctions (or branching points), and vessel length. Processing of cerebrovascular data was done by personnel blinded to the sex or age groups.

### 2.3 MRI acquisition, processing, and analysis

High-resolution T2-RARE images were acquired using an 11.7 T Bruker Avance Instrument. T2-RARE images (repetition time/echo time: 6482 ms/49.3 ms, 28 × 0.55 mm slices) were collected on a 200 × 155 matrix with a 2 × 1.55 cm field of view. Brain tissue was extracted from the skull case of T2RARE scans using 3D Pulse-Coupled Neural Networks (PCNN3D) in Matlab R2017a (RRID:SCR_001622, MathWorks). Extraction masks were reviewed and adjusted using ITK-SNAP (version 3.6.0, RRID:SCR_002010; Yushkevich et al., 2006) and scans went through N4 Bias field correction (Tustison et al., 2010). A modified bilateral Australian Mouse Brain Mapping Consortium Atlas (Ullmann et al., 2013) was fit to each individual animal, and regional labels were applied with Advanced Normalization Tools (ANTs, RRID:SCR_004757; Avants et al., 2011). An example of a T2RARE scan with brain region labels is shown in Supplementary Figure 1. Whole-brain and cerebrum (excluding olfactory bulbs and cerebellum) volumes were exported from the extraction masks using the ITK Snap software. Brain regional volumes were extracted for 40 bilateral regions (see Supplementary Table 1) as raw volumes (mm^3^) and normalized to the cerebrum of each individual mouse. Heatmaps of brain regional volumes were created with Qlucore Omics Explorer (Version 3.9.23, RRID:SCR_027904, Qlucore AB, Lund, Sweden).

### 2.4 Statistical analysis

GraphPad Prism 9 software (RRID:SCR_000306, GraphPad Prism, San Diego, CA) was used to perform all statistical analyses. A two-way ANOVA followed by Sidak’s multiple comparisons test was used for vessel analysis, whole brain, cerebrum and regional brain volumes. Graphs are presented as mean ± SD. Outlier removal for the vessel characteristic metrics was performed using a 1.5*IQR test (interquartile range) cutoff on vessel density, this led to the exclusion of two 6-month-old females, one 18-month-old female and one 26-month-old male for all metrics.

## 3 Results

### 3.1 Body weight, whole brain and cerebrum volumes across the C57BL/6N lifespan

**Figure 1:**
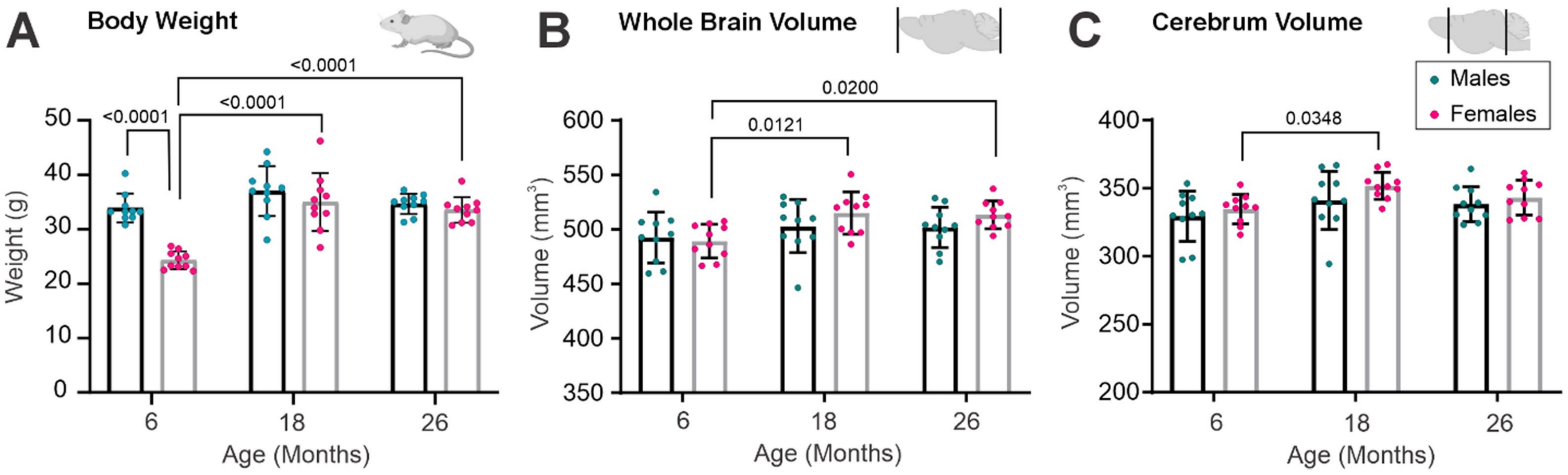
Lifespan evolution of **(A)** body weight, **(B)** whole brain, and **(C)** cerebrum volumes in C57BL/6N mice across sex. Statistics: 2way ANOVA followed by Sidak’s multiple comparisons test, *n* = 10 per group. Biorender.com was used to generate this figure.

The body weight of male C57BL/6N mice remained relatively constant between 6, 18 and 26 months of age (33.9 ± 2.6 g, 37.0 ± 4.6 g and 34.6 ± 1.8 g, respectively, Figure 1A). Female body weight was significantly lower than males at 6 months (24.3 ± 1.6 g vs. 33.9 ± 2.6 g) and then increased significantly at 18 and 26 months (35.0 ± 5.3 g and 33.5 ± 2.4 g, respectively, Figure 1A). Significant age and sex effects were detected by 2way ANOVA analysis with *p* < 0.0001 for both factors.

MRI acquisitions were used to assess whole and regional brain volumes. We found sex-specific changes in the whole brain and cerebrum volumes of normal aging C57BL/6N mice. Globally, significant sex differences in whole brain (*p* = 0.0072) and cerebrum (*p* = 0.0131) volumes were detected in 2way ANOVA analysis. With increasing age, whole brain and cerebrum volumes for males stayed relatively constant (Figures 1B,C). In contrast, female mice aged 18- and 26-month-old exhibited significantly larger whole brain volumes than 6-month-old females (6 vs. 18 vs. 26 months: 489.2 ± 15.5 vs. 515.0 ± 19.4 vs. 513.3 ± 12.8 mm^3^, Figure 1B). Similarly, female cerebrum volumes also reported a significant increase between 6 and 18 months of age (334.6 ± 10.9 vs. 351.7 ± 9.9 mm^3^, Figure 1C). No significant sex-dependent differences (male vs. female) were observed at 6, 18, or 26 months for whole brain and cerebrum volumes. Thus, female mice brains increased with age but not males. Interestingly, we found a significant effect of age on lateral ventricle volume but no effect of sex (2way ANOVA, *p* = 0.0095 and *p* = 0.5529, respectively). The volume is significantly increased in females between the ages of 18 and 26 months (*p* = 0.0334), with no other significant differences being detected (data not shown).

**Figure 2:**
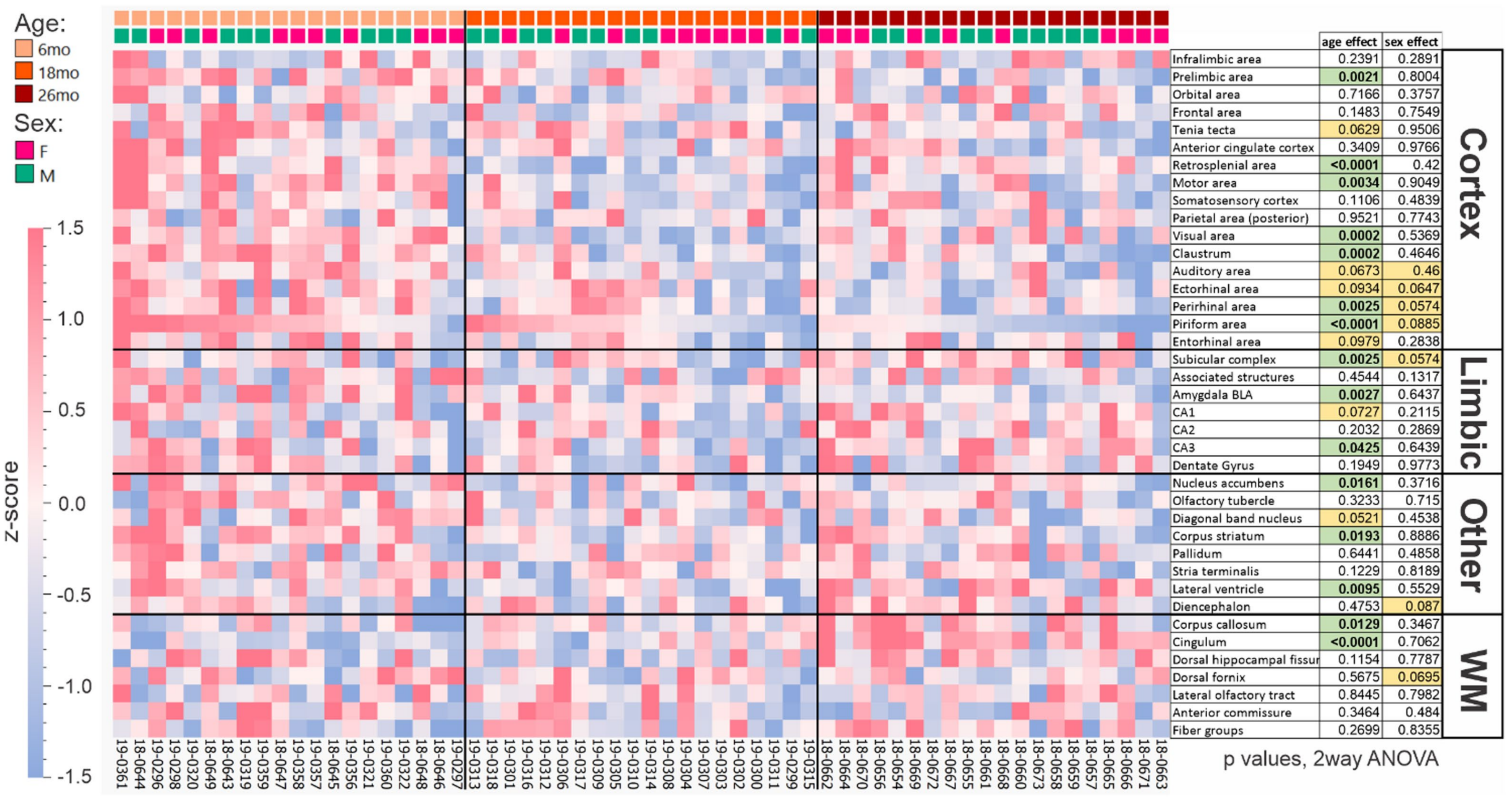
Heatmap of normalized brain region volumes (% of cerebrum volume) in C57BL/6N mice sorted by age. Each column is an individual mouse and each row is a brain region volume. Note the trend of decreasing “red” over time in cortical regions but an opposite increasing “red” in white matter regions. Volumes for each region were normalized over all mice using *z*-score normalization and is shown using a color scheme based on *z*-score distribution from −1.5 to 1.5, *n* = 10 per group. The table on the right shows *p* values from 2way ANOVA corresponding to age and sex effects. Green cells highlight *p* value <0.05, yellow cells highlight *p* < 0.1.

Regional brain volume changes were sorted by broad groups: cortex, limbic, white matter and other regions for each individual mouse. The heatmap illustrates a global decrease in cortical volumes but an increased white matter volume with age (Figure 2).

### 3.2 Cortical volumes decrease with normal aging

We next examined how specific brain regions changed during normal aging in male and female C57BL/6N mice. We used a modified (bilateral segmentation) atlas derived from the AMBMC (Ullmann et al., 2013), as detailed in Supplementary Table 1. Given the temporal change in cerebrum volumes with age, particularly in female mice, we reported brain regions normalized to the cerebrum volume, where normalization was performed individually for each mouse. Raw (mm^3^) and corrected (% of cerebrum) regional volumes for all 40 regions are provided in tabular format (Supplementary Table 2).

**Figure 3:**
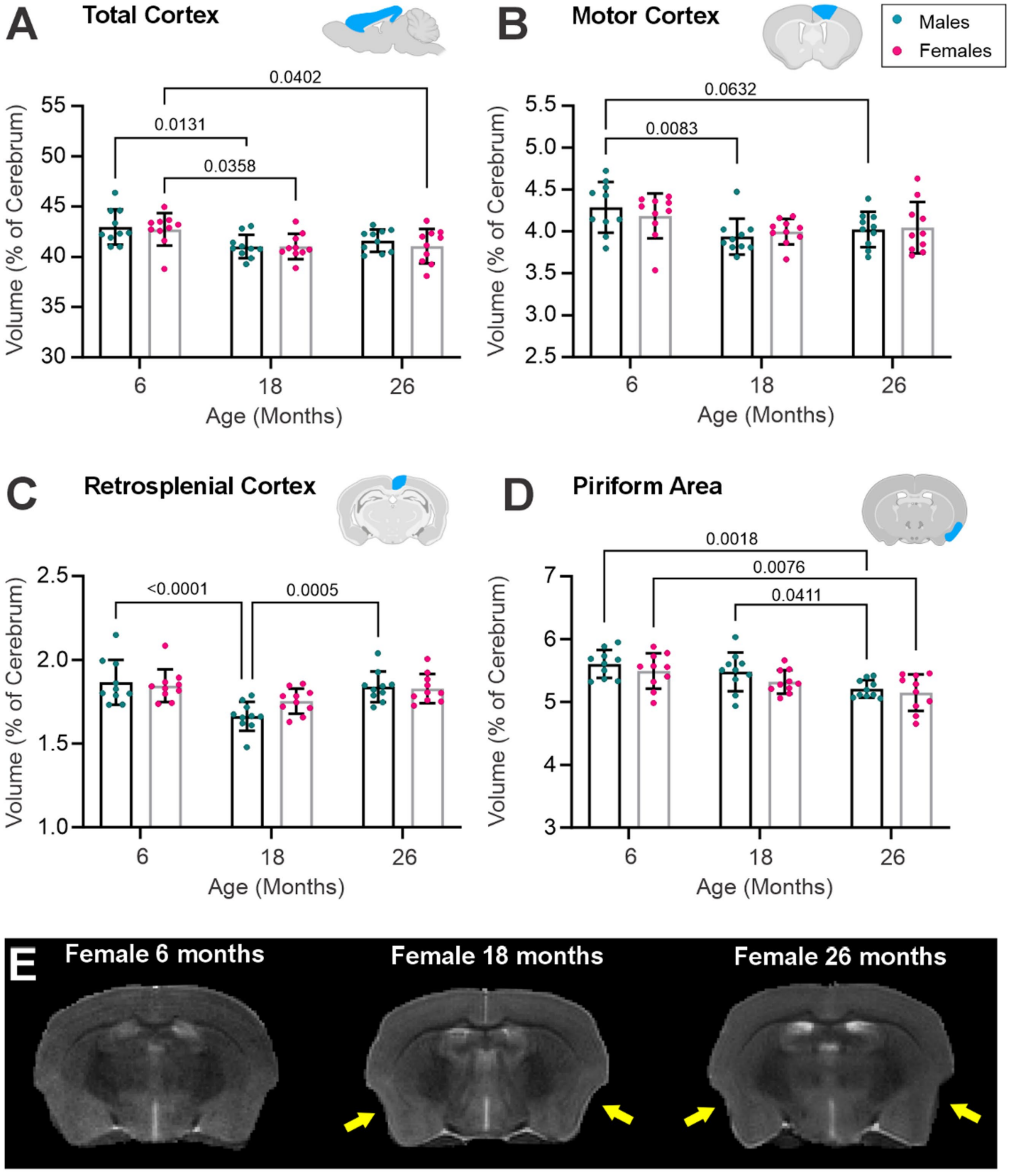
Cortical volume changes in aging C57BL/6N mice. Volumes of **(A)** aggregated total, **(B)** motor, **(C)** retrosplenial, and **(D)** piriform cortices expressed as % of cerebrum volumes in male and female C57BL/6N mice illustrate age-related decreases. **(E)** T2RARE images from 6-, 18-, and 26-month-old females at Bregma ~ − 1.0 mm. Yellow arrows identify visible areas of cortical thinning. Statistics: 2way ANOVA followed by Sidak’s multiple comparisons, *n* = 10 per group. Biorender.com was used to generate this figure.

When all cortical regions were aggregated, we observed a significant decrease in normalized cortical volumes with increasing age in both sexes (2way ANOVA, *p* = 0.0005). Male and female total cortex volumes were significantly lower at 18 months compared to 6 months of age (6-month vs. 18-month, males: 43.0 ± 1.7% vs. 41.0 ± 1.2% and females: 42.7 ± 1.6 vs. 41.0 ± 1.3%, Figure 3A). The significant cortical volume decrease at 18 months in female mice was maintained in females between 6 and 26 months of age (42.7 ± 1.6% vs. 41.0 ± 1.7%, Figure 3A) but not in male mice.

We next examined which cortical regions contributed to the age-related decline. In the motor cortex, we found a significant age effect (2way ANOVA, *p* = 0.0034) but no sex differences (*p* = 0.905). Males at 18 and 26 months had smaller motor cortex volumes compared to 6 months (6 months vs. 18 months vs. 26 months: 4.29 ± 0.30% vs. 3.94 ± 0.21% vs. 4.02 ± 0.21%, Figure 3B). This was significant between 6 and 18 months (*p* = 0.0083) and trending between 6 and 26 months (*p* = 0.0632, Figure 3B). Motor cortex volumes were not significantly different between ages in female mice (Figure 3B).

In the retrosplenial cortex, volumetric changes were significantly affected by age (2way ANOVA, *p* < 0.0001). The retrosplenial cortex volume of males was significantly lower at 18 months compared to 6 and 26 months of age (6 months vs. 18 months vs. 26 months: 1.87 ± 0.13% vs. 1.66 ± 0.09% vs. 1.84 ± 0.09%, Figure 3C). In females, no significant changes in retrosplenial cortex volume were detected (Figure 3C). Piriform cortex volumes in male and female mice were significantly decreased with age (2way ANOVA, *p* < 0.0001) but not altered by sex (*p* = 0.0885). Males showed lower piriform area volumes with age (6 months vs. 18 months vs. 26 months: 5.61 ± 0.22% vs. 5.48 ± 0.31% vs. 5.21 ± 0.14%, Figure 3D), as well as females (6 months vs. 18 months vs. 26 months: 5.50 ± 0.28% vs. 5.32 ± 0.19% vs. 5.15 ± 0.29%, Figure 3D).

In summary, total, motor, retrosplenial, and piriform cortices were not significantly altered between males and females at any of the ages examined (Figures 3A–D). T2RARE scans visually displayed cortical thinning between 6- to 18- and 26-month-old (shown for females in Figure 3E) which aligns with findings of decreased piriform area volumes with age (Figure 3D).

### 3.3 Evolution of limbic volumes during normal aging

**Figure 4:**
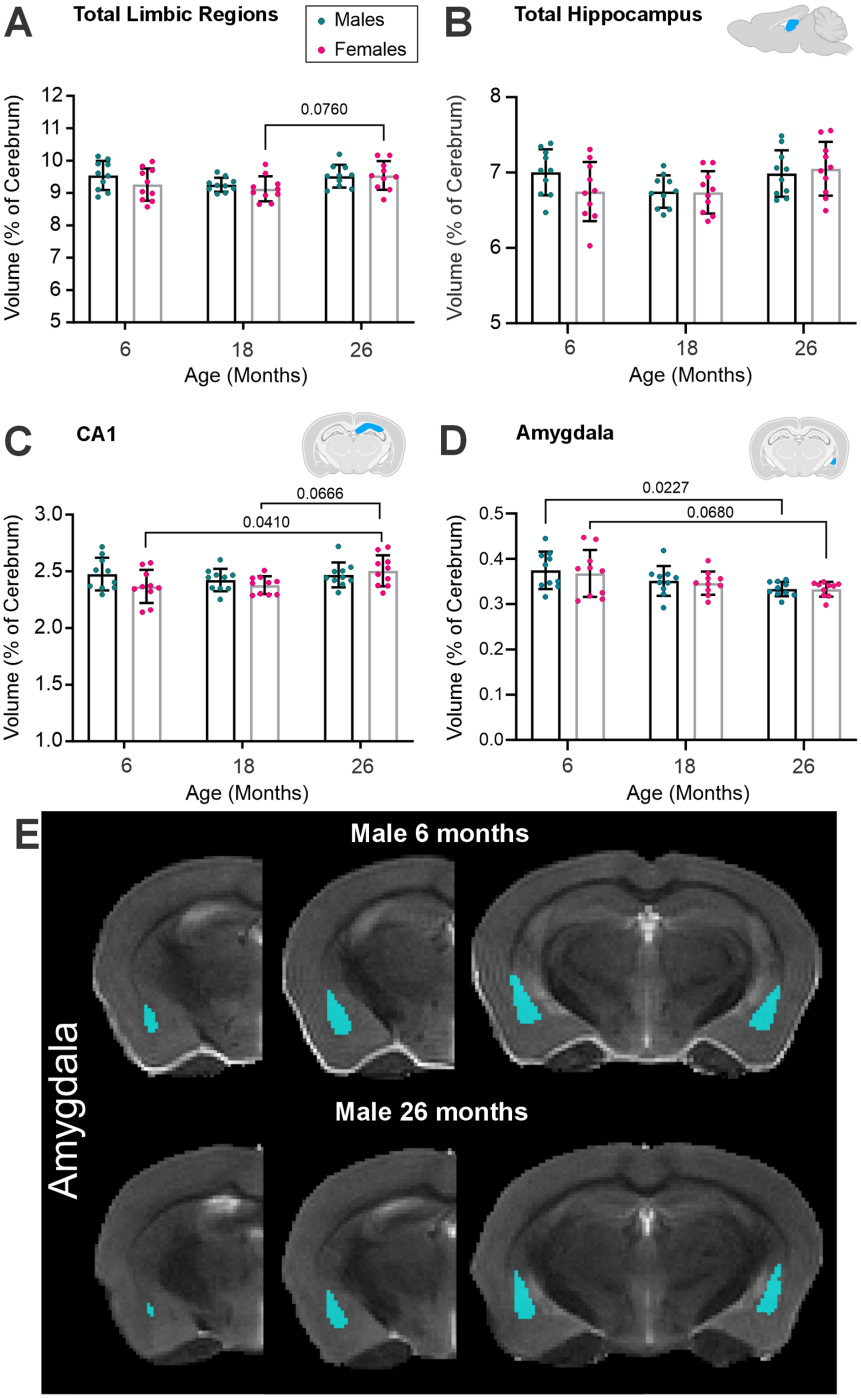
Changes in regional limbic volumes in C57BL/6N mice with age and sex. Volumes of **(A)** all limbic regions combined, **(B)** hippocampus, **(C)** CA1 subregion, and **(D)** amygdala volumes in male and female C57BL/6N mice. **(E)** Amygdala was the only limbic region with age-related decreases, as shown in T2RARE images from males at 6 and 26 months of age. Statistics: 2-way ANOVA followed by Sidak’s multiple comparisons, *n* = 10 per group. Biorender.com was used to generate this figure.

Human limbic regions are recognized to be vulnerable to aging (Bogdanovic et al., 2025) and normative aging results in progressive volumetric declines with age (Gohel et al., 2025). Our initial analysis combined all limbic regions in aggregate (see list in Supplementary Table 1) and we observed that limbic volumes were significantly impacted by age (2way ANOVA, *p* = 0.0336). No sex differences were detected at any age, albeit there was a trending increase in total limbic volume in females between 18 and 26 months of age (9.13 ± 0.39% and 9.54 ± 0.44%, *p* = 0.0760, Figure 4A). No differences were observed in males between any ages (Figure 4A).

Hippocampal volumes were impacted by age (2way ANOVA, *p* = 0.0269), but there were no significant differences between ages or sexes (Figure 4B). Hippocampal subregions, such as CA1 had a trending effect of age (2way ANOVA, *p* = 0.0727, Figure 4C). Amygdala volumes were significantly decreased with age (2way ANOVA, *p* = 0.0027) with both males and females reporting a significant decrease in amygdala volumes between 6 and 26 months of age (6 months vs. 26 months: males: 0.37 ± 0.04% vs. 0.33 ± 0.02%, females: 0.37 ± 0.05% vs. 0.33 ± 0.02%, Figure 4D, 4E). Total limbic, hippocampus, CA1 subregion, and amygdala volumes showed no significant differences between sexes at any age.

### 3.4 White matter volumes increase with age

**Figure 5:**
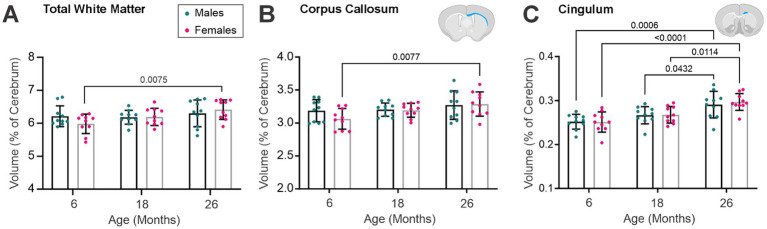
Changes in white matter volumes in C57BL/6N mice with age and sex. Volumes of **(A)** total white matter, **(B)** corpus callosum, and **(C)** cingulum volumes in male and female C57BL/6N mice. Statistics: 2-way ANOVA followed by Sidak’s multiple comparisons, *n* = 10 per group. Biorender.com was used to generate this figure.

White matter volumes and myelin content in humans are impacted by age (Bethlehem et al., 2022; Groh and Simons, 2025). All white matter volumes combined (total, see list in Supplementary Table 1), corpus callosum, and cingulum volumes were all significantly increased with age (2way ANOVA, *p* = 0.0295, *p* = 0.0129 and *p* < 0.0001, respectively) but with no sex effects (2way ANOVA, *p* = 0.2247, *p* = 0.3467 and *p* = 0.7062, respectively) (Figures 5A–C). Interestingly, we found that 26-month-old females had a significantly larger total white matter volume than 6-month-old females (4 vs. 24-month: 5.98 ± 0.30% vs. 6.41 ± 0.30%, *p* = 0.0075, Figure 5A). In the corpus callosum a similar significant increase was observed (6 vs. 26 months, 3.06 ± 0.16% vs. 3.29 ± 0.18%, *p* = 0.0077, Figure 5B). Cingulum volumes were also increased with age in both males and females with significant differences between 6 and 26, and between 18 and 26 months of age (Figure 5C). Thus, opposite to limbic regional decrements, white matter structures increased with age.

### 3.5 Angioarchitecture of the cortical surface

**Figure 6:**
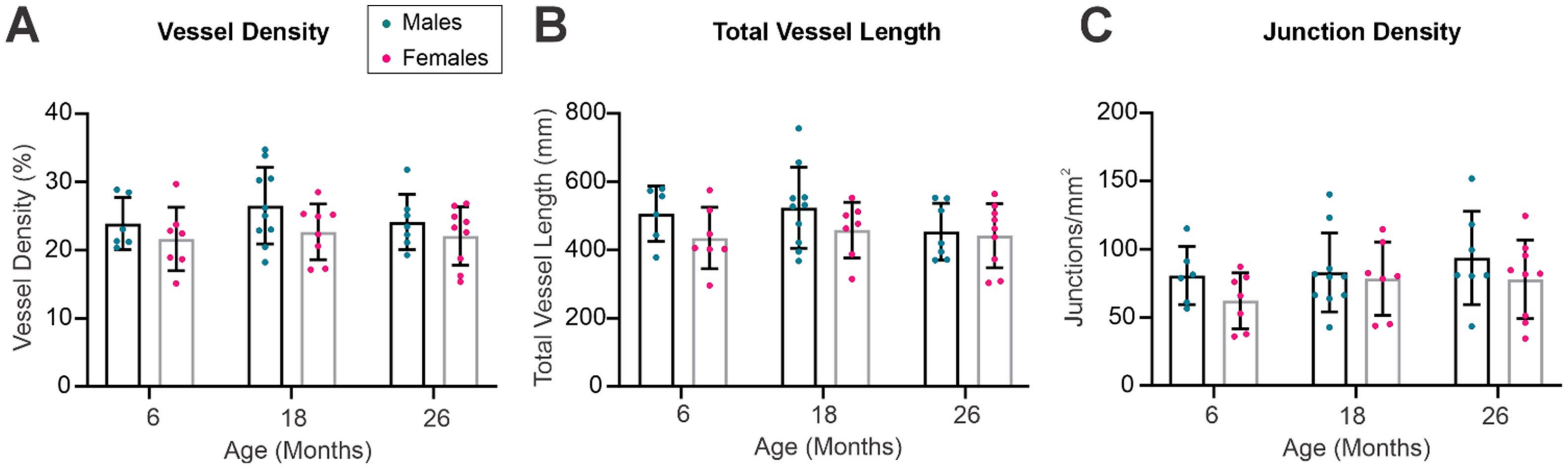
Cortical surface vessel characteristics in male and female C57BL/6N with increasing age. **(A)** Vessel density, **(B)** total vessel length, and **(C)** junction density were not significantly different between groups. Statistics: 2way ANOVA followed by Sidak’s multiple comparisons, *n* = 6–10 per group (6 months: *n* = 6 males and 7 females, 18 months: *n* = 10 males and 7 females, 26 months: *n* = 7 males and 9 females).

Cerebrovascular morphology of the cortical brain surface was assessed using our vessel painting technique (Salehi et al., 2018). Vessel density on the cortical surface was globally altered by sex with females having lower density (2way ANOVA, *p* = 0.0441), but not by age (2way ANOVA, *p* = 0.5846, Figure 6A). Similarly, total vessel length showed a trend toward an effect of sex with lower values in females (2way ANOVA, *p* = 0.0885), but no effect of age (2way ANOVA, *p* = 0.4396, Figure 6B). Junction density (number of branching points per mm^2^) exhibited no effects of sex (2way ANOVA, *p* = 0.1254) or age (2way ANOVA, *p* = 0.3881, Figure 6C). While there were vascular changes over time by age and sex, there were no significant differences.

## 4 Discussion

Normative aging is the normal and gradual physical, cognitive and biological changes that occur during healthy aging which is distinct from pathological processes such as Alzheimer’s disease where changes are accelerated and more pronounced (Fjell et al., 2014). Brain morphology is known to change with age in humans (Courchesne et al., 2000; Bethlehem et al., 2022) with volumetric declines across widespread brain regions with concomitant increases in ventricular size (Courchesne et al., 2000; Armstrong et al., 2019; Bethlehem et al., 2022). Aging males face steeper volumetric declines than females across most brain regions; importantly these declines are non-linear (Armstrong et al., 2019; Bethlehem et al., 2022). These longitudinal declines in cognitively healthy males are faster across whole brain volumes, total gray and white matter (Armstrong et al., 2019). Interestingly, the rates of volume loss in humans do not differ between sexes for amygdala, entorhinal cortex or temporal lobe, and white matter (Armstrong et al., 2019). Thus, in the aging human brain males are more affected in specific regions while in others (i.e., amygdala) the rates of decline are similar between sexes.

Preclinical studies, however, most often use mice for which brain morphology changes through normative aging have been sparsely explored. Here, we sought to analyze brain region morphology and cortical surface vasculature across three adult ages in males and females C57BL/6N, a strain widely used in aging research. We report the following novel findings: (1) total cortex, piriform area, and amygdala volumes are decreased with age in both sexes, (2) motor and retrosplenial cortices volumes are altered only in males across the lifespan, (3) whole brain, cerebrum, CA1, total white matter and corpus callosum volumes are increased with age only in females, (4) cingulum volumes are increased with age in both males and females across the lifespan, and (5) no significant differences in cortical surface vasculature were found across ages nor between sexes. Broadly, except for body weight, we found no significant differences between males and females within specific age groups.

Our findings of decreased C57BL/6N amygdala volume mirror the clinical literature, where decreased amygdala volumes are described during normal aging in humans (Jack et al., 1997; Mu et al., 1999). Volumetric decrements in the amygdala volumes have not been previously reported in mouse MRI studies, although a histological study found increased axonal fragments in the amygdala of aged (21 to 25-month-old) male mice compared to adults (8-month-old) (Von Bohlen und Halbach and Unsicker, 2002). The authors reported decreased neuronal density within the basolateral amygdala which may underlie the reduced amygdala volumes during aging.

Other regions with decreased volumes across age for both males and females were the total cortex and piriform area. Decreases in cortical thickness and grey matter loss are well described during human aging (Courchesne et al., 2000; Armstrong et al., 2019; Bethlehem et al., 2022). A mouse study found no significant differences in the cerebral cortex volume (in mm^3^) of male C57BL/6 mice from 2 to 22 months of age (Clifford et al., 2023), consistent with our non-normalized results (ie. in mm^3^, see Supplementary Table 2). In contrast, another study assessing cerebral cortex volumes changes from 6 to 14 months of age found no significant differences (Maheswaran et al., 2009). In our study, the motor cortex volumes were only decreased in males. Interestingly, the retrosplenial cortex temporally evolved differently in males during aging with decreased volumes between 6 and 18 months of age followed by an increased volume at 26 months of age. This progressive reduction in volumes which later increased has also been observed in male Wistar rats where the retrosplenial, cingulate and entorhinal cortices, all referred to as higher-order areas, were shown to increase with age (Gull et al., 2023).

Previous mouse studies have shown whole brain volume increases during aging, in early adulthood and mature stages (von Kienlin et al., 2005; Maheswaran et al., 2009; Jullienne et al., 2023). This is at odds with human data where decrements in brain volume appear after adolescence (Courchesne et al., 2000; Scahill et al., 2003; Bethlehem et al., 2022). Our volumetric findings mostly align with previous studies (von Kienlin et al., 2005; Maheswaran et al., 2009; Hill et al., 2020; Clifford et al., 2023; Jullienne et al., 2023) as we observed increased whole brain and cerebrum volumes in females with age, and no overt whole brain and cerebrum volumes alterations in male C57BL/6N from 6 to 26 months of age.

Unlike grey matter that decreases linearly during human normative aging, white matter has been shown to remain steady during adulthood, reaching a plateau around 30–40 years of age and decreasing after about 50–60 years (Courchesne et al., 2000; Raz and Rodrigue, 2006; Yang et al., 2016; Bethlehem et al., 2022; Groh and Simons, 2025). In contrast to the human studies, we found increased total white matter and corpus callosum volumes, but only in females. White matter tract volumes have been reported to increase in a cross-sectional study of male C57BL/6 mice from 2 to 22 months (Clifford et al., 2023). We also showed in a recent longitudinal study that the corpus callosum volumes of male mice remained essentially stable across the lifespan, between 5 and 18 months of age (Obenaus et al., 2025).

The cingulum is another major white matter structure connecting limbic structures between frontal and posterior cortices. Clinical studies assessing cingulum volume are lacking but Pagani reported decreased cingulum volumes during human aging (Pagani et al., 2008). Rather than assessing the cingulum volumes, most clinical studies focus on structural properties derived from diffusion tensor imaging (DTI), such as fractional anisotropy (FA) and mean diffusivity (MD) (Ezzati et al., 2016; Jang et al., 2016; Edde et al., 2020; Aghamohammadi-Sereshki et al., 2022). For example, there is decreased FA and increased MD in the heathy aging cingulum consistent with putative reductions in fiber density (Ezzati et al., 2016; Jang et al., 2016; Aghamohammadi-Sereshki et al., 2022). In other studies, MD and FA were shown to correlate with verbal fluency in older individuals (Edde et al., 2020). Contrary to current human findings, our MRI-derived results show that cingulum volume is significantly increased in male and female mice across their lifespan. Table 1 compares our findings with human MRI data.

**Table 1:** Comparison between our murine study and published human MRI studies.

Brain region	Volume evolution during aging	
	Our study	Human MRI studies
Whole brain/cerebrum	Increased in females	Increased (Courchesne et al., 2000; Scahill et al., 2003; Bethlehem et al., 2022)
Total cortex	Decreased in males and females	Decreased (Courchesne et al., 2000; Armstrong et al., 2019; Bethlehem et al., 2022)
White Matter	Increased in females	Decreased (Courchesne et al., 2000; Raz and Rodrigue, 2006; Yang et al., 2016; Bethlehem et al., 2022; Groh and Simons, 2025)
Cingulum	Increased in males and females	Decreased (Pagani et al., 2008)
Amygdala	Decreased in males and females	Decreased (Jack et al., 1997; Mu et al., 1999)
Hippocampus	No significant changes	Decreased (Jack et al., 1997; Mu et al., 1999; Scahill et al., 2003)

Most preclinical aging mouse studies focus on males and their regional brain volume (Maheswaran et al., 2009; Clifford et al., 2023; Obenaus et al., 2025), with a smaller number of publications reporting on females only (von Kienlin et al., 2005; Hill et al., 2020). This complicates assessments of sex differences and comparisons with our study due to differences in age, genetic background, and acquisition methods. Although our study reported that aging differentially affects brain regions between male and female C57BL/6N mice, we did not find any notable sex differences at any specific age group we investigated.

Given the volumetric alterations we found, we then assessed the vascular characteristics of the cortical surface. Notably, we did not find any significant effects of aging or sex on vessels from the cortical surface. In a previous study of vessel painting in C57BL/6J mice, we found an increase in vascular features (vessel density, junction density, and vessel length) in males between the ages of 4 and 12 months. No differences were detected in females during this aging epoch (Jullienne et al., 2023). Several other studies have demonstrated a decrease in vascular density during aging in several brain regions of rats, mice and humans with most of these studies using histology or other *ex vivo* techniques [reviewed in Riddle et al. (2003) and Brown and Thore (2011)]. Cerebrovascular loss was also detected *in vivo* using contrast-enhanced magnetic resonance angiography (MRA) and apparent cerebral blood volume in female C57BL/6N mice from 2 to 26 months of age (Hill et al., 2020). These discrepancies in findings could be explained by species, brain regions, acquisition and analysis methods, and range of ages used in the studies. As reviewed by Riddle and colleagues (Riddle et al., 2003) and more recently by Jin (2019), aging-related changes in microvessel density have been reported to either decrease, increase, or remain unchanged for both humans and rodents. Some studies even showed a biphasic modulation of brain microvessels with an increase in vessel density between young and adult subjects, followed by a decrease in old (28-month-old) rats (Wilkinson et al., 1981), and in humans (Hunziker et al., 1979).

Our study had several limitations. The brain (via MRI) and vascular morphology were assessed *ex vivo* in a cross-sectional manner. Using an *in vivo* longitudinal experimental design could decrease subject-specific random effects. Moreover, using *in vivo* MRI would allow the possibility of tracking cerebrovascular measures such as cerebral blood flow and cerebral blood volume across the lifespan, as others have reported (Wei et al., 2020; Vasilkovska et al., 2024). Methods such as optical coherence tomography would allow longitudinal tracking of cerebrovascular morphology, topology and function at the cortical surface (Walek et al., 2023). Ultrasound localization microscopy, an emerging imaging modality that achieves visualization of microvasculature in deep tissues with high spatial resolution could also be very informative (Nyul-Toth et al., 2024; Wang et al., 2025; Negri et al., 2026).

In summary, our study provides a broad framework for understanding and considering differential patterns of brain regional changes during aging of healthy male and female C57BL/6N mice. These studies are critically important considering the large number of transgenic mouse models related to aging and Alzheimer’s disease.

## Data Availability

The original contributions presented in the study are included in the article/Supplementary material, further inquiries can be directed to the corresponding author.
