# Supplementary material for: Regional brain and cerebrovasculature morphology during normative aging in male and female C57BL/6N mice
Source: Front Aging Neurosci. 2026 Jun 16;18:1852741. doi: 10.3389/fnagi.2026.1852741 (PMC13351089; doi:10.3389/fnagi.2026.1852741)
Supplement: Supplementary file 1 [file Data_Sheet_1.docx]

Regional Brain Morphology and Cerebrovasculature of Male and Female C57BL/6N Mice during Normative Aging

**Amandine Jullienne ^1, 2^, Tannoz Norouzi ^1^, Erik Behringer ^3^, Andre Obenaus ^1, 2,*^.**

^1^Pediatrics Department, University of California Irvine, Irvine, CA, USA

^2^Present Address: Biomedical Sciences Division, School of Medicine, University of California Riverside, Riverside, CA, USA

^3^Basic Sciences Department, Loma Linda University, Loma Linda, CA, USA

*** Correspondence:**Andre Obenaus

University of California Riverside

Div. of Biomedical Sciences

207 SOM Research Building

Riverside, CA 92521

andre.obenaus@medsch.ucr.edu

**Supplemental Materials**

**Supplemental Table 1: Categorize brain regions derived from our modified Australian Mouse Brain Mapping Consortium atlas** (Ullmann et al., 2013)

| Cortex | Infralimbic area |
| --- | --- |
|  | Prelimbic area |
|  | Orbital area |
|  | Frontal area |
|  | Tenia tecta |
|  | Anterior cingulate cortex |
|  | Retrosplenial area |
|  | Motor area |
|  | Somatosensory cortex |
|  | Parietal area (posterior) |
|  | Visual area |
|  | Claustrum |
|  | Auditory area |
|  | Ectorhinal area |
|  | Perirhinal area |
|  | Piriform area |
|  | Entorhinal area |
| Limbic | Subicular complex |
|  | Associated structures |
|  | Amygdala BLA |
|  | CA1 |
|  | CA2 |
|  | CA3 |
|  | Dentate Gyrus |
| White Matter | Corpus callosum |
|  | Cingulum |
|  | Dorsal hippocampal fissure |
|  | Dorsal fornix |
|  | Lateral olfactory tract |
|  | Anterior commissure |
|  | Fiber groups |
| Other | Nucleus accumbens |
|  | Olfactory tubercle |
|  | Diagonal band nucleus |
|  | Corpus striatum |
|  | Pallidum |
|  | Stria terminalis |
|  | Lateral ventricle |
|  | Diencephalon |
|  | Cerebellum |

**Supplemental Table 2. Regional Brain Sizes (mm^3^)**

|  |  | Males 6 months |  | Females 6 months |  | Males 18 months |  | Females 18 months |  | Males 26 months |  | Females 26 months |  |
| --- | --- | --- | --- | --- | --- | --- | --- | --- | --- | --- | --- | --- | --- |
|  |  | Average | SEM | Average | SEM | Average | SEM | Average | SEM | Average | SEM | Average | SEM |
| **Region** | **Category** | Size (mm^3) | Size (mm^3) | Size (mm^3) | Size (mm^3) | Size (mm^3) | Size (mm^3) | Size (mm^3) | Size (mm^3) | Size (mm^3) | Size (mm^3) | Size (mm^3) | Size (mm^3) |
| Infralimbic_area | cortex | 0.564 | 0.024 | 0.483 | 0.019 | 0.521 | 0.019 | 0.456 | 0.011 | 0.581 | 0.024 | 0.591 | 0.019 |
| Prelimbic_area | cortex | 2.299 | 0.012 | 2.201 | 0.018 | 2.218 | 0.011 | 2.358 | 0.023 | 2.087 | 0.016 | 2.213 | 0.013 |
| Orbital_area | cortex | 6.766 | 0.061 | 6.843 | 0.041 | 7.024 | 0.057 | 7.260 | 0.036 | 7.153 | 0.033 | 6.898 | 0.038 |
| Frontal_area | cortex | 6.588 | 0.077 | 6.807 | 0.061 | 6.716 | 0.063 | 6.696 | 0.065 | 6.810 | 0.083 | 7.167 | 0.057 |
| Tenia_tecta | cortex | 1.229 | 0.017 | 1.265 | 0.013 | 1.246 | 0.014 | 1.398 | 0.014 | 1.258 | 0.009 | 1.153 | 0.016 |
| Anterior_cingulate_cortex | cortex | 4.192 | 0.026 | 4.097 | 0.037 | 4.250 | 0.036 | 4.490 | 0.038 | 4.147 | 0.040 | 4.235 | 0.037 |
| Retrosplenial_area | cortex | 6.034 | 0.048 | 6.021 | 0.035 | 5.672 | 0.046 | 6.170 | 0.036 | 6.218 | 0.032 | 6.267 | 0.025 |
| Motor_area | cortex | 13.845 | 0.081 | 13.657 | 0.102 | 13.410 | 0.074 | 14.058 | 0.065 | 13.601 | 0.060 | 13.842 | 0.067 |
| Somatosensory_cortex | cortex | 31.590 | 0.135 | 32.073 | 0.205 | 32.041 | 0.150 | 33.833 | 0.202 | 32.616 | 0.266 | 32.897 | 0.109 |
| Parietal_area_(posterior) | cortex | 1.039 | 0.025 | 0.940 | 0.025 | 0.980 | 0.015 | 1.095 | 0.028 | 1.029 | 0.030 | 1.022 | 0.009 |
| Visual_area | cortex | 13.386 | 0.073 | 13.894 | 0.066 | 13.400 | 0.083 | 13.866 | 0.085 | 14.344 | 0.132 | 13.752 | 0.069 |
| Claustrum | cortex | 8.850 | 0.057 | 8.962 | 0.039 | 8.874 | 0.053 | 9.066 | 0.059 | 8.775 | 0.057 | 8.683 | 0.053 |
| Auditory_area | cortex | 7.380 | 0.062 | 7.365 | 0.030 | 7.488 | 0.062 | 7.698 | 0.070 | 7.428 | 0.100 | 7.319 | 0.035 |
| Ectorhinal_area | cortex | 2.726 | 0.024 | 2.676 | 0.022 | 2.822 | 0.031 | 2.723 | 0.029 | 2.702 | 0.037 | 2.628 | 0.017 |
| Perirhinal_area | cortex | 2.553 | 0.020 | 2.432 | 0.013 | 2.510 | 0.028 | 2.534 | 0.022 | 2.430 | 0.020 | 2.397 | 0.012 |
| Piriform_area | cortex | 18.122 | 0.081 | 17.917 | 0.085 | 18.649 | 0.075 | 18.718 | 0.102 | 17.621 | 0.086 | 17.648 | 0.088 |
| Entorhinal_area | cortex | 11.712 | 0.098 | 11.723 | 0.065 | 11.920 | 0.095 | 11.938 | 0.113 | 12.054 | 0.090 | 11.968 | 0.089 |
| Subicular_complex | limbic | 3.813 | 0.049 | 3.895 | 0.035 | 4.148 | 0.042 | 3.994 | 0.052 | 4.135 | 0.032 | 4.270 | 0.033 |
| Associated_structures | limbic | 3.177 | 0.024 | 3.094 | 0.042 | 3.198 | 0.036 | 3.209 | 0.042 | 3.295 | 0.035 | 3.119 | 0.020 |
| Amygdala_BLA | limbic | 1.214 | 0.017 | 1.197 | 0.015 | 1.196 | 0.012 | 1.217 | 0.010 | 1.125 | 0.003 | 1.142 | 0.006 |
| CA1 | limbic | 8.004 | 0.050 | 7.717 | 0.043 | 8.263 | 0.058 | 8.364 | 0.042 | 8.347 | 0.035 | 8.583 | 0.038 |
| CA2 | limbic | 1.057 | 0.016 | 0.991 | 0.019 | 1.195 | 0.021 | 1.057 | 0.014 | 1.098 | 0.013 | 1.232 | 0.016 |
| CA3 | limbic | 6.057 | 0.042 | 5.932 | 0.051 | 6.076 | 0.049 | 6.382 | 0.048 | 6.450 | 0.036 | 6.480 | 0.041 |
| Dentate_Gyrus | limbic | 7.526 | 0.045 | 7.362 | 0.053 | 7.454 | 0.032 | 7.899 | 0.044 | 7.729 | 0.053 | 7.871 | 0.047 |
| Nucleus_accumbens | other | 4.476 | 0.046 | 4.568 | 0.023 | 4.550 | 0.033 | 4.599 | 0.030 | 4.574 | 0.017 | 4.460 | 0.015 |
| Olfactory_tubercle | other | 4.066 | 0.044 | 4.104 | 0.027 | 4.175 | 0.027 | 4.180 | 0.032 | 4.102 | 0.036 | 4.193 | 0.019 |
| Diagonal_band_nucleus | other | 1.588 | 0.023 | 1.634 | 0.015 | 1.624 | 0.018 | 1.634 | 0.012 | 1.503 | 0.018 | 1.621 | 0.014 |
| Corpus_striatum | other | 19.688 | 0.075 | 19.400 | 0.104 | 19.708 | 0.098 | 20.511 | 0.065 | 19.647 | 0.064 | 20.149 | 0.071 |
| Pallidum | other | 3.917 | 0.022 | 3.791 | 0.029 | 3.983 | 0.031 | 4.102 | 0.021 | 3.951 | 0.020 | 4.058 | 0.020 |
| Stria_terminalis | other | 0.970 | 0.008 | 1.040 | 0.008 | 1.022 | 0.014 | 1.017 | 0.014 | 0.972 | 0.014 | 0.984 | 0.013 |
| Lateral_ventricle | other | 2.817 | 0.020 | 2.732 | 0.031 | 2.740 | 0.023 | 2.764 | 0.027 | 2.946 | 0.034 | 3.026 | 0.030 |
| Diencephalon | other | 25.383 | 0.116 | 24.619 | 0.146 | 26.484 | 0.148 | 27.163 | 0.145 | 26.709 | 0.114 | 27.075 | 0.064 |
| Cerebellum | other | 59.405 | 0.418 | 57.920 | 0.301 | 60.471 | 0.307 | 60.559 | 0.406 | 57.618 | 0.316 | 59.967 | 0.158 |
| Corpus_callosum | wm | 10.315 | 0.085 | 9.983 | 0.041 | 10.920 | 0.072 | 11.226 | 0.044 | 11.064 | 0.075 | 11.268 | 0.061 |
| Cingulum | wm | 0.816 | 0.007 | 0.820 | 0.008 | 0.909 | 0.009 | 0.941 | 0.006 | 0.981 | 0.009 | 1.019 | 0.007 |
| Dorsal_hippocampal_fissure | wm | 1.393 | 0.036 | 1.313 | 0.025 | 1.296 | 0.017 | 1.369 | 0.026 | 1.418 | 0.023 | 1.564 | 0.024 |
| Dorsal_fornix | wm | 0.031 | 0.002 | 0.040 | 0.001 | 0.028 | 0.001 | 0.042 | 0.001 | 0.036 | 0.001 | 0.031 | 0.001 |
| Lateral_olfactory_tract | wm | 0.437 | 0.011 | 0.431 | 0.011 | 0.476 | 0.006 | 0.477 | 0.009 | 0.454 | 0.007 | 0.470 | 0.008 |
| Anterior_commissure | wm | 0.984 | 0.026 | 0.925 | 0.018 | 0.953 | 0.024 | 0.945 | 0.019 | 0.903 | 0.020 | 0.906 | 0.023 |
| Fiber_groups | wm | 6.141 | 0.046 | 6.005 | 0.047 | 6.499 | 0.058 | 6.771 | 0.044 | 6.438 | 0.038 | 6.740 | 0.043 |

**Supplemental Table 3. Regional Brain Sizes (% of Brain Volume)**

|  |  | Males 6 months |  | Females 6 months |  | Males 18 months |  | Females 18 months |  | Males 26 months |  | Females 26 months |  |
| --- | --- | --- | --- | --- | --- | --- | --- | --- | --- | --- | --- | --- | --- |
|  |  | Average | SEM | Average | SEM | Average | SEM | Average | SEM | Average | SEM | Average | SEM |
| **Region** | **Category** | Size (%) | Size (%) | Size (%) | Size (%) | Size (%) | Size (%) | Size (%) | Size (%) | Size (%) | Size (%) | Size (%) | Size (%) |
| Infralimbic_area | cortex | 0.174 | 0.007 | 0.147 | 0.006 | 0.152 | 0.005 | 0.130 | 0.004 | 0.166 | 0.007 | 0.172 | 0.006 |
| Prelimbic_area | cortex | 0.711 | 0.004 | 0.675 | 0.007 | 0.652 | 0.006 | 0.670 | 0.008 | 0.628 | 0.006 | 0.646 | 0.006 |
| Orbital_area | cortex | 2.092 | 0.018 | 2.097 | 0.013 | 2.062 | 0.017 | 2.065 | 0.018 | 2.142 | 0.016 | 2.014 | 0.015 |
| Frontal_area | cortex | 2.042 | 0.029 | 2.090 | 0.023 | 1.978 | 0.027 | 1.902 | 0.021 | 2.001 | 0.024 | 2.091 | 0.021 |
| Tenia_tecta | cortex | 0.380 | 0.006 | 0.388 | 0.005 | 0.367 | 0.005 | 0.397 | 0.005 | 0.380 | 0.004 | 0.336 | 0.005 |
| Anterior_cingulate_cortex | cortex | 1.298 | 0.011 | 1.256 | 0.012 | 1.246 | 0.007 | 1.276 | 0.013 | 1.237 | 0.012 | 1.235 | 0.011 |
| Retrosplenial_area | cortex | 1.866 | 0.016 | 1.846 | 0.015 | 1.663 | 0.010 | 1.754 | 0.011 | 1.848 | 0.015 | 1.829 | 0.010 |
| Motor_area | cortex | 4.287 | 0.031 | 4.185 | 0.031 | 3.938 | 0.022 | 3.997 | 0.021 | 4.070 | 0.023 | 4.044 | 0.033 |
| Somatosensory_cortex | cortex | 9.773 | 0.044 | 9.829 | 0.054 | 9.408 | 0.037 | 9.615 | 0.040 | 9.465 | 0.047 | 9.601 | 0.048 |
| Parietal_area_(posterior) | cortex | 0.323 | 0.010 | 0.288 | 0.009 | 0.287 | 0.007 | 0.310 | 0.012 | 0.290 | 0.009 | 0.298 | 0.005 |
| Visual_area | cortex | 4.139 | 0.019 | 4.258 | 0.019 | 3.930 | 0.015 | 3.941 | 0.022 | 4.173 | 0.031 | 4.013 | 0.025 |
| Claustrum | cortex | 2.736 | 0.016 | 2.748 | 0.016 | 2.606 | 0.015 | 2.576 | 0.022 | 2.594 | 0.015 | 2.533 | 0.019 |
| Auditory_area | cortex | 2.281 | 0.017 | 2.258 | 0.015 | 2.194 | 0.011 | 2.187 | 0.017 | 2.114 | 0.023 | 2.136 | 0.014 |
| Ectorhinal_area | cortex | 0.844 | 0.010 | 0.821 | 0.010 | 0.827 | 0.009 | 0.773 | 0.008 | 0.771 | 0.011 | 0.767 | 0.010 |
| Perirhinal_area | cortex | 0.791 | 0.009 | 0.746 | 0.008 | 0.735 | 0.008 | 0.720 | 0.007 | 0.706 | 0.006 | 0.699 | 0.007 |
| Piriform_area | cortex | 5.606 | 0.024 | 5.495 | 0.030 | 5.482 | 0.033 | 5.320 | 0.022 | 5.203 | 0.018 | 5.150 | 0.030 |
| Entorhinal_area | cortex | 3.623 | 0.031 | 3.597 | 0.030 | 3.495 | 0.023 | 3.391 | 0.029 | 3.501 | 0.022 | 3.491 | 0.029 |
| Subicular_complex | limbic | 1.179 | 0.016 | 1.195 | 0.015 | 1.214 | 0.011 | 1.133 | 0.015 | 1.230 | 0.013 | 1.246 | 0.013 |
| Associated_structures | limbic | 0.984 | 0.009 | 0.950 | 0.016 | 0.939 | 0.011 | 0.913 | 0.012 | 0.972 | 0.010 | 0.911 | 0.010 |
| Amygdala_BLA | limbic | 0.375 | 0.004 | 0.368 | 0.005 | 0.351 | 0.004 | 0.346 | 0.003 | 0.338 | 0.003 | 0.333 | 0.002 |
| CA1 | limbic | 2.476 | 0.016 | 2.367 | 0.015 | 2.423 | 0.012 | 2.378 | 0.012 | 2.494 | 0.016 | 2.505 | 0.016 |
| CA2 | limbic | 0.328 | 0.006 | 0.303 | 0.006 | 0.349 | 0.006 | 0.300 | 0.004 | 0.338 | 0.005 | 0.359 | 0.006 |
| CA3 | limbic | 1.873 | 0.014 | 1.819 | 0.017 | 1.783 | 0.014 | 1.813 | 0.013 | 1.936 | 0.017 | 1.889 | 0.014 |
| Dentate_Gyrus | limbic | 2.327 | 0.012 | 2.258 | 0.020 | 2.192 | 0.017 | 2.246 | 0.014 | 2.297 | 0.018 | 2.297 | 0.018 |
| Nucleus_accumbens | other | 1.383 | 0.012 | 1.401 | 0.010 | 1.335 | 0.009 | 1.309 | 0.011 | 1.362 | 0.005 | 1.302 | 0.008 |
| Olfactory_tubercle | other | 1.257 | 0.013 | 1.258 | 0.009 | 1.230 | 0.014 | 1.189 | 0.010 | 1.238 | 0.013 | 1.224 | 0.010 |
| Diagonal_band_nucleus | other | 0.489 | 0.006 | 0.501 | 0.005 | 0.477 | 0.006 | 0.465 | 0.004 | 0.439 | 0.005 | 0.473 | 0.006 |
| Corpus_striatum | other | 6.090 | 0.022 | 5.950 | 0.037 | 5.785 | 0.021 | 5.833 | 0.016 | 5.912 | 0.031 | 5.879 | 0.029 |
| Pallidum | other | 1.211 | 0.006 | 1.162 | 0.010 | 1.167 | 0.007 | 1.167 | 0.007 | 1.191 | 0.010 | 1.184 | 0.007 |
| Stria_terminalis | other | 0.301 | 0.003 | 0.319 | 0.003 | 0.300 | 0.004 | 0.289 | 0.004 | 0.294 | 0.004 | 0.287 | 0.004 |
| Lateral_ventricle | other | 0.871 | 0.007 | 0.838 | 0.012 | 0.804 | 0.009 | 0.786 | 0.010 | 0.891 | 0.011 | 0.884 | 0.011 |
| Diencephalon | other | 7.852 | 0.032 | 7.549 | 0.046 | 7.771 | 0.026 | 7.726 | 0.042 | 7.950 | 0.039 | 7.900 | 0.029 |
| Cerebellum | other | 18.361 | 0.096 | 17.764 | 0.108 | 17.744 | 0.043 | 17.224 | 0.114 | 17.109 | 0.091 | 17.490 | 0.051 |
| Corpus_callosum | wm | 3.185 | 0.018 | 3.062 | 0.017 | 3.202 | 0.012 | 3.192 | 0.012 | 3.336 | 0.023 | 3.287 | 0.020 |
| Cingulum | wm | 0.252 | 0.002 | 0.251 | 0.002 | 0.266 | 0.002 | 0.268 | 0.002 | 0.301 | 0.003 | 0.297 | 0.002 |
| Dorsal_hippocampal_fissure | wm | 0.430 | 0.012 | 0.403 | 0.010 | 0.382 | 0.010 | 0.390 | 0.011 | 0.443 | 0.008 | 0.456 | 0.009 |
| Dorsal_fornix | wm | 0.010 | 0.001 | 0.012 | 0.000 | 0.008 | 0.000 | 0.012 | 0.000 | 0.011 | 0.000 | 0.010 | 0.000 |
| Lateral_olfactory_tract | wm | 0.135 | 0.003 | 0.132 | 0.003 | 0.140 | 0.002 | 0.136 | 0.003 | 0.143 | 0.003 | 0.137 | 0.002 |
| Anterior_commissure | wm | 0.302 | 0.007 | 0.283 | 0.006 | 0.279 | 0.007 | 0.269 | 0.006 | 0.265 | 0.006 | 0.263 | 0.006 |
| Fiber_groups | wm | 1.899 | 0.014 | 1.841 | 0.015 | 1.905 | 0.014 | 1.926 | 0.014 | 1.951 | 0.017 | 1.965 | 0.013 |
