# Supplementary material for: Regional brain and cerebrovasculature morphology during normative aging in male and female C57BL/6N mice
Source: Front Aging Neurosci. 2026 Jun 16;18:1852741. doi: 10.3389/fnagi.2026.1852741 (PMC13351089; doi:10.3389/fnagi.2026.1852741)
Supplement: Supplementary file 3 [file Supplementary_file_1.docx]

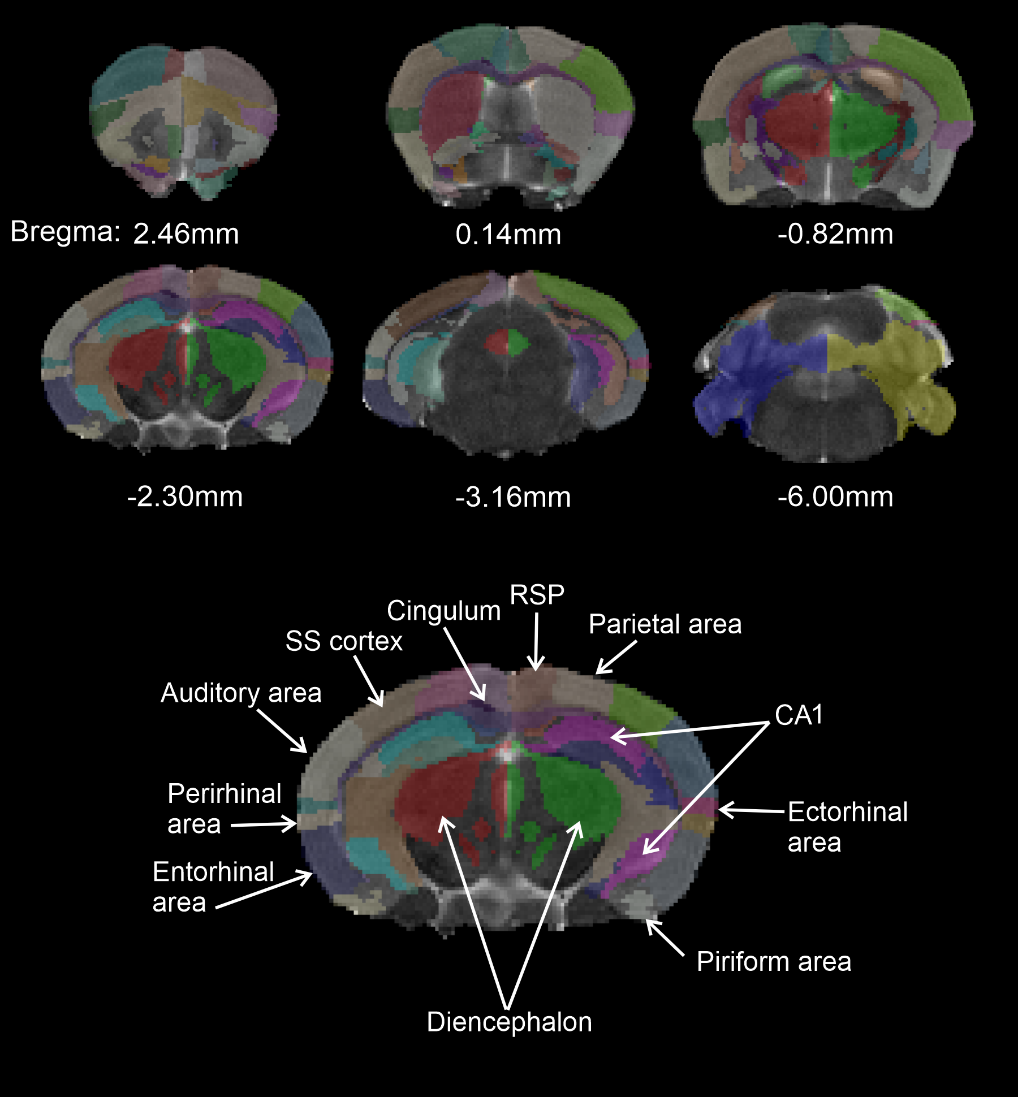


Supplement Figure 1. Example of brain region labels from the Australian Mouse Brain Mapping Consortium Atlas (Ullmann et al., 2013) registered to one mouse. This atlas was used in our automated pipeline for extracting brain regions. Abbreviations: SS-somatosensory, RSP-retrosplenial.
